# Myeloid-derived suppressor cells exhibit distinct characteristics in bone marrow and blood of individuals with diffuse large B-cell lymphoma

**DOI:** 10.3389/fmed.2024.1515097

**Published:** 2025-01-29

**Authors:** Paris Efstratiou, Athina Damianaki, Aglaia Kavidopoulou, Polymnia Ioannidou, Effrosyni Markaki, Ioannis Moysis Skianis, Electra Tsagliotis, Vasilia Kaliafentaki, Angelos Mattheakakis, Maria Ximeri, Eleftherios Manouras, Matthieu Lavigne, Panayotis Verginis, Christina Kalpadakis

**Affiliations:** ^1^Laboratory of Hematology, Division of Laboratory Medicine, School of Medicine, University of Crete and University Hospital of Heraklion, Heraklion, Greece; ^2^Laboratory of Immune Regulation and Tolerance, Division of Basic Sciences, School of Medicine, University of Crete, Heraklion, Greece; ^3^Institute of Molecular Biology and Biotechnology (IMBB), Foundation for Research and Technology-Hellas (FORTH), Heraklion, Greece

**Keywords:** DLBCL, MDSCs, bone marrow, lymphomas, transcriptomic analysis

## Abstract

Antitumor immune surveillance is the key feature of tumour progression and response to treatment in various malignancies, such as lymphomas. Myeloid derived suppressor cells (MDSCs) are bone marrow (BM)-derived cells with potent suppressive properties, implicated in T cell inhibition and tumour dissemination. In Diffuse Large B-cell Lymphoma (DLBCL), circulating MDSCs constitute the immunosuppressive tumor microenvironment, while the contribution of BM MDSCs in disease pathogenesis remains elusive. In the present study we aimed to evaluate both the frequencies as well as the molecular signatures of MDSCs in blood and BM from newly diagnosed DLBCL patients prior to treatment initiation and from age matched healthy donors. Circulating levels of total, monocytic (M-) and polymorphonuclear (PMN-) MDSCs were found increased in DLBCL compared to healthy control, while in DLBCL patients the BM MDSCs were significantly increased compared to blood. Transcriptomic analysis revealed significantly different molecular fingerprints to characterize circulating and BM M-MDSCs, implying that MDSCs exhibit their function with distinct mechanisms depending on the anatomical compartment. Despite that MDSC frequencies did not demonstrate any significant correlation with disease characteristics and outcome, our findings propose that gene expression profiling should be evaluated for their potential prognostic impact. Overall, the findings presented here, provide new insights in the immunosuppressive networks that operate in DLBCL and importantly propose new molecular mechanisms expressed by BM MDSCs which may be explored therapeutically.

## Introduction

Diffuse large B-cell lymphomas (DLBCL) represent the most common form of lymphomas characterized by an aggressive clinical course and short survival if left untreated (1). Immunochemotherapy with the combination of the anti-CD20 monoclonal antibody rituximab along with CHOP chemotherapy (cyclophosphamide, doxorubicin, vincristine and prednisone) has been considered as the established first line treatment (2). Recently, the addition of polatuzumab vedotin, an antibody–drug conjugate targeting CD79b, to the RCHP treatment improved the responses and the progression-free survival (PFS) (3). Despite the improvement in first line treatment, still 30–40% of patients will relapse or be refractory to first line treatment (4). The outcome for relapsed/refractory disease is particularly poor, with a median overall survival of less than 3 years (5–7). The introduction of T-cell mediated treatments such as CART-cells and bispecific antibodies has revolutionized the treatment of relapsed/refractory DLBCL (8–11). However, only a minority of individuals with DLBCL (~40%) will eventually respond to these agents. Therefore, it is apparent that unappreciated mechanisms of tumor immune evasion exist, and their delineation is urgently needed in order to design rational therapies for DLBCL.

Numerous prognostic markers have been evaluated and used in DLBCL, such as the International Prognostic Index (IPI) and the revised R-IPI, which are among the most commonly and widely used systems for risk stratification (12–15). However, due to the high heterogeneity of the disease with the dismal prognosis in almost 30% of patients, the search for novel prognostic factors and biomarkers to reliably predict therapy responses remain an unmet need. The disease complexity is highlighted by the genomic analysis, which revealed a highly diverse landscape since DLBCL can be categorized as germinal center B-cell-like (GCB), activated B-cell–like (ABC), and unclassifiable DLBCL, based on the cell of origin (16–21). The above categorization is of prognostic significance, since patients with GCB-DLBCL display better overall survival (OS) compared to ABC-subtype. Besides the aforementioned prognostic markers, recent findings indicate that the immune profile of the patients to influences the therapeutic outcomes. Immunosuppressive cells, such as regulatory T cells (Tregs) and myeloid-derived suppressor cells (MDSCs) present both in the tumor microenvironment (TME), as well as in blood have been implicated in immune escape and dissemination of the tumor (22). However, in DLBCL, most studies focus on the phenotypic characterization of such cells, while their functional importance remains elusive. Delineating the precise mechanisms via which the immunosuppressive cells imprint in the development of the hostile TME and the impairment of immunotherapy response will provide insights into the design of novel immunotherapeutic regimens for DLBCL patients.

Myeloid-derived suppressor cells (MDSCs) constitute a heterogeneous population of immature cells of myeloid origin, which, in steady-state conditions, reside in the bone marrow (BM). At the same time, upon inflammatory triggering, they are released in the periphery and exhibit potent immunosuppressive functions. Phenotypically, human MDSCs are divided into cells of monocytic origin termed M-MDSCs and cells of granulocytic origin termed PMN-MDSCs. Despite the extensive heterogeneity and the diverse markers used to describe human MDSCs in the literature, a consensus was recently proposed for the identification of MDSC subsets by flow cytometry (23). Thus, M-MDSCs are identified as CD11b^+^CD14^+^ HLA-DR^–/low^CD15^−^CD33^+^ while PMN-MDSCs as CD11b^+^CD14^−^ HLA-DR^−/low^ CD15^+^CD33^+^. MDSCs markedly expand during solid tumor development and heavily accumulate in the tumor microenvironment facilitating tumor growth and also impeding immunotherapy responses (24). In addition, MDSCs have been shown to regulate various inflammatory conditions, including autoimmune and infectious diseases, graft-versus-host disease and chronic inflammation (25–27).

Furthermore, MDSCs have been reported to be significantly enriched in blood and TME of hematological malignancies, such as multiple myeloma (MM), acute myeloid leukemia (AML), acute promyelocytic leukemia (APL), B-cell acute lymphoblastic leukemia (B-ALL) myelodysplastic syndromes (MDS), and in chronic myeloid leukemia (CML), as extensively reviewed elsewhere (28–32), and to associate with high tumor burden, adverse clinical features, and outcome. Specifically, in DLBCL, several studies have provided ample evidence for the expansion of M-MDSCs as well as PMN-MDSCs in blood, which correlated with disease activity or the Revised-International Prognostic Index (R-IPI) and the histologic subtype. Attempts were also made to evaluate the prognostic significance of MDSCs in DLBCL individuals regarding response to treatment and outcome (33). Despite the aforementioned knowledge, the functional properties of BM MDSCs remain completely unexplored. The BM is the compartment where MDSCs are generated and reside, and therefore, it would be of interest to identify mechanisms that are either shared between peripheral and BM MDSCs or are uniquely operated by BM cells and contribute to DLBCL.

In the present study, we focus on BM and peripheral MDSCs in DLBCL patients of identifying mechanisms that influence the outcome of the disease and may dictate therapeutic outcomes. Our findings show that MDSCs in DLBCL patients are increased both in peripheral blood and in the BM compared to the respective sites in healthy individuals. Transcriptomic analysis revealed distinct signatures between M-MDSCs in the periphery and BM of DLBCL patients, suggesting that depending on the anatomic site, MDSCs exert differently their immunosuppressive properties.

## Materials and methods

### Patients

The DLBCL cohort consisted of 33 newly diagnosed patients recruited through the Haematology Clinic at the University General Hospital of Heraklion, Greece and an age-matched group of 12 healthy individuals. Diagnosis was based on the 5^th^ WHO criteria. Eligible healthy donors had no history of autoimmune disease or immunosuppression due to a known disease or medication. Bone marrow was obtained from healthy volunteers during total hip arthroplasty at the Orthopedic Clinic of the University General Hospital of Heraklion, Greece.

All DLBCL patients were treated homogeneously with the RCHOP immunochemotherapy combination. Exclusion criteria were infections, autoimmune diseases, or prior steroid treatment. Demographics, disease characteristics, and outcomes were collected from the patients’ medical files after signing informed consent.

The study was approved by the Ethics Committee of the Institutional Review Board of the University General Hospital of Heraklion, Greece and all samples were obtained with informed written consent per the Declaration of Helsinki.

### Flow cytometry analysis in whole blood and bone marrow samples

Peripheral blood and BM samples were collected into tubes containing EDTA, and mononuclear cells (PBMCs and BMMCs) were isolated over a density gradient using Ficoll-Paque Plus (GE Healthcare) within 24 h of sample collection. Following density gradient centrifugation, at least 1 million single cells per sample were stained with fluorochrome-coupled antibodies. The monoclonal antibodies used for labelling of samples were: ECD-labelled anti-HLA-DR (Immu-357), PC7-labeled anti-CD33 (D3HL60.251), APC-labeled anti-CD14 (RM052), FITC-labeled anti-CD11b (94), PE-labeled anti-CD15 (80H5), all antibodies from Beckman Coulter-Immunotech.

**Figure 1:**
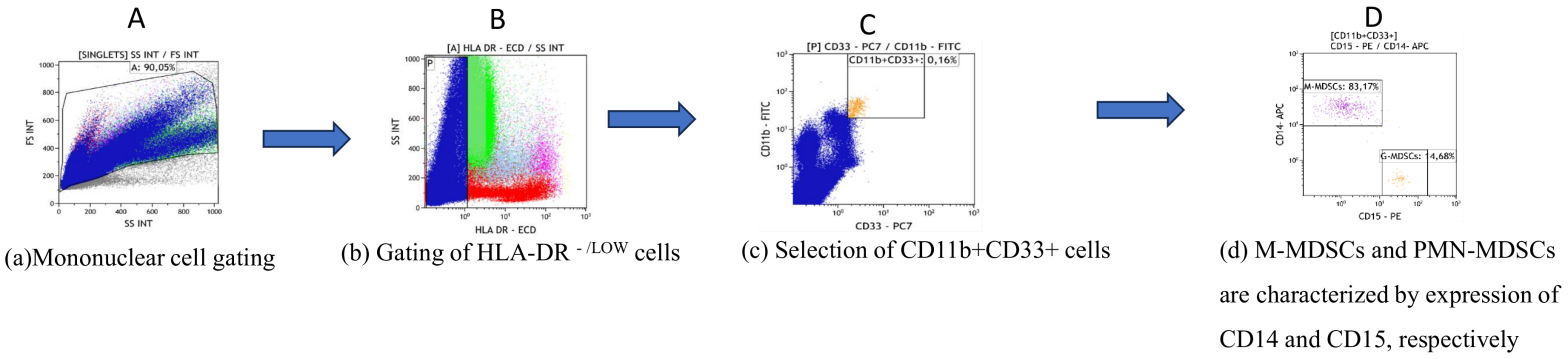
Gating strategy for the phenotypic characterization of MDSCs and subsets in blood and bone marrow. **(A)** The main gate is plotted on side scatter (SSC) area versus forward scatter (FSC) after excluding dead cells and debris. **(B)** HLA-DR -/LOW cells were then gated and **(C)** analysed for CD33 and CD11b expression. Total MDSCs were defined as CD33-positive, CD11b-positive cells and **(D)** further divided into M-MDSCs characterized by CD14 expression and PMN-MDSCs expressing CD15. Analysis was performed using KALUZA software version 10.

Total MDSCs, monocytic MDSCs (M-MDSCs) and granulocytic myeloid derived suppressor cells (PMN-MDSCs) were measured by flow cytometry using the NAVIOS cytometry system (Beckmann Coulter) and analysed using KALUZA software (Beckmann Coulter). Gating strategy is shown in Figure 1. Briefly, total MDSCs were assessed as CD11b^+^CD33^+^ HLADR^low/−^, while M-MDSCs were gated as CD11b^+^CD33^+^ HLADR^low/−^CD14+ CD15− and PMN-MDSCs as CD11b + CD33+ HLA-DR^low/−^CD14− CD15+. Cell sorting of MDSCs was performed under aseptic conditions using 70-micron nozzle with a BD FACS Aria III (BD Biosciences).

### RNA isolation and 3′ RNA sequencing

RNA from FACS-sorted M-MDSCs was isolated using Nucleospin RNA XS kit (Macherey-Nagel), according to the manufacturer’s instructions. The quality and quantity of isolated RNA were measured using 2,100 Bioanalyser (Agilent) and RNA 6000 Pico Kit reagents (Agilent). RNA samples with RNA integrity number (RIN) > 7 were used for library construction using the 3′ mRNA-Seq Library Prep Kit FWD for Illumina (QuantSeq-LEXOGEN) as per the manufacturer’s instructions. Amplification was controlled to obtain optimal unbiased libraries across samples by assessing the number of cycles (ranging from 20 to 24) required by qPCR. DNA High Sensitivity Kit for bioanalyzer was used to assess the quantity and quality of libraries, according to the manufacturer’s instructions (Agilent). Libraries were multiplexed and sequenced on an Illumina Nextseq 500 at the genomics facility of IMBB FORTH according to the manufacturer’s instructions.

### Statistical analysis

Statistical analysis was performed using unpaired Student’s *t*-test in GraphPad Prism v5 software (GraphPad Prism, RRID: SCR_002798). Data are presented as means ± S.E.M. *p*-value < 0.05 was considered as indicative of statistical significance. All *p*-values and n are reported in the figure legends.

## Results

### Clinical characteristics of DLBCL patients

From November 2019 to September 2023, 33 patients diagnosed with DLBCL were included in the present study. The main clinical findings are presented in Table 1. The median age was 71 (range 26–88) with a male predominance (20/33). Eighteen patients had limited stage (I + II) disease and low and low intermediate International Prognostic Index (IPI). Among the 25 cases where the cell of origin was available, 10 had a germinal center type (GCB) DLBCL. All patients were treated with RCHOP and 21 achieved a complete remission at the end of treatment, while 4 had a partial response and 8 had disease progression. At a median follow up time of 15.5 months, 7 patients have died of disease progression.

**Table 1:** Main clinical characteristics of the 33 DLBCL patients.

Clinical features	ALL (*n* = 33)
Sex (male)	20 (60%)
Age (median) (range)	71 (26–88)
Cell of origin (COO)	
GCB subtype	10 (30%)
Non-GCB subtype	15 (46%)
Double Hit	1 (3%)
Unknown	7 (21%)
Ann Arbor stage	
I-II	18 (54%)
III-IV	15 (46%)
LDH elevated	17 (51%)
IPI	
Low risk (IPI 0-2)	18 (54%)
High risk (IPI 3-4)	15 (46%)

GCB, germinal center B-cell like; LDH, Lactate dehydrogenase; IPI, international prognostic index.

### Increased frequencies of MDSCs in blood and BM of DLBCL individuals

**Figure 2:**
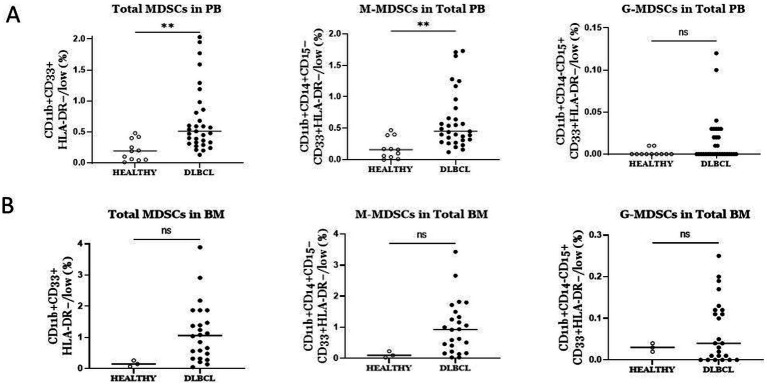
Comparison of the frequencies of total MDSCs and subtypes between DLBCL patients and Healthy individuals in blood **(A)** and bone marrow. **(A)** Total and M-MDSCs in blood were significantly elevated in DLBCL individuals compared to healthy subjects. No significant difference was observed in PMN-MDSCs between the two groups. **(B)** No difference was observed in total MDSCs and subtypes in bone marrow of DLBCL individuals compared to healthy subjects. ** *p* < 0.01, ns: no significant.

Considering the critical immunosuppressive role of MDSCs, we sought to determine whether their frequencies were altered in blood of our DLBCL cohort (*n* = 31) compared to healthy controls (*n* = 11), by flow cytometry analysis. The gating strategy that followed to characterize the total MDSCs as well as the M-MDSCs and PMN-MDSCs is shown in Figure 1. To this end, total MDSCs were significantly increased in PB of DLBCL individuals compared to healthy subjects (Figure 2A). This was accompanied by a marked increase of M-MDSCs, while PMN-MDSCs, although there was a trend of increase in DLBCL, did not reach statistical significance (Figure 2A).

Since MDSCs are BM-derived cells, we asked whether their frequencies present differences in the BM of DLBCL compared to healthy individuals. As expected, total MDSCs, as well as M-MDSCs and PMN-MDSCs, were expanded in the BM of DLBCL patients (*n* = 24), however, statistical analysis did not yield significance, most likely due to the low number of BM samples from healthy individuals (*n* = 3) (Figure 2B). Collectively, these findings confirm previous studies which demonstrate significant increase of MDSCs in PB of DLBCL patients and importantly, indicate a prominent expansion of MDSCs in the BM of these individuals.

### Marked enrichment of MDSCs in BM compared to PB in DLBCL individuals

**Figure 3:**
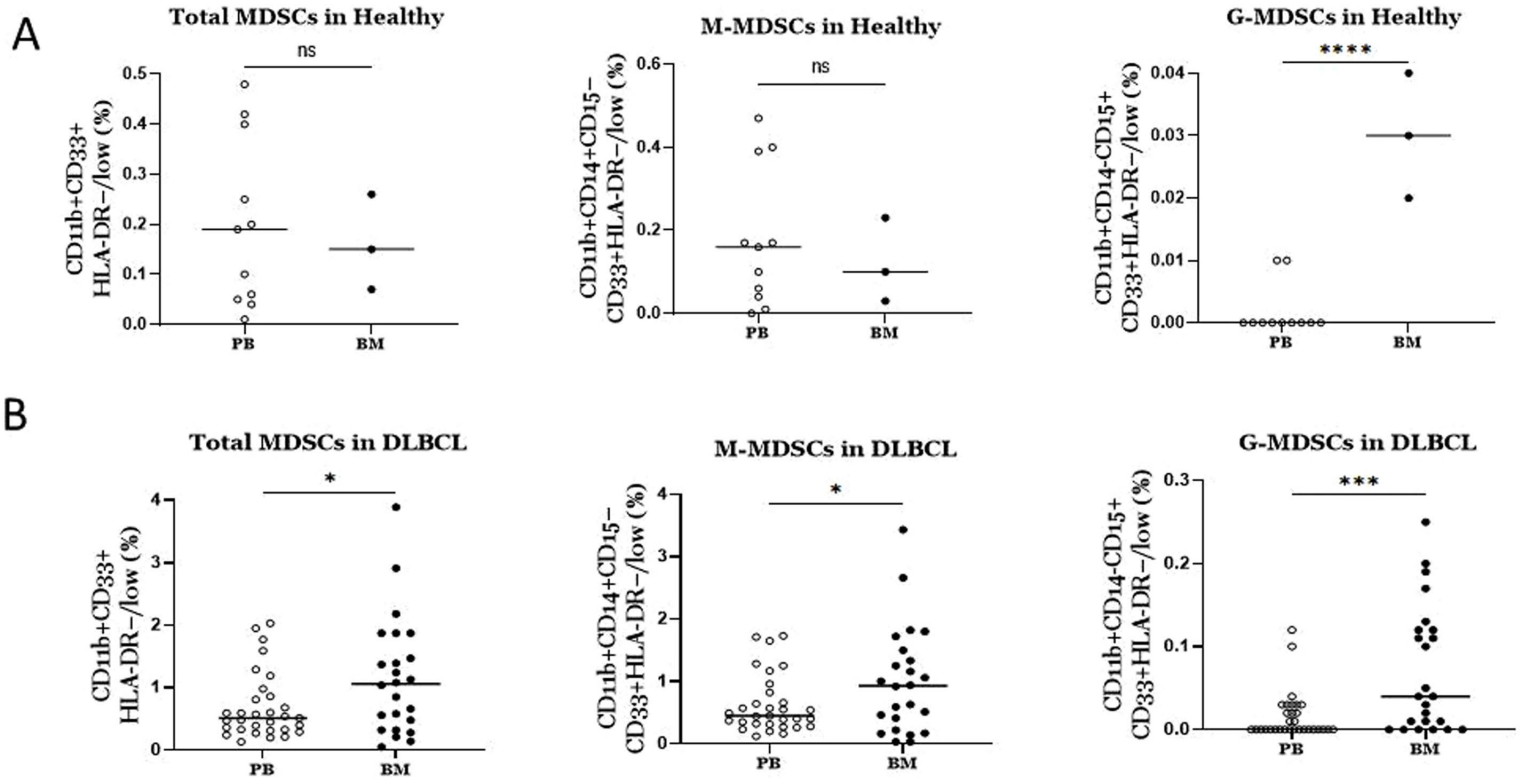
Comparison of MDSCs frequencies between blood and BM in DLBCL patients and healthy individuals **(A)** In DLBCL patients MDSCs (total and subtypes) were significantly elevated in bone marrow compared to blood. **(B)** In Healthy individuals only PMN-MDSCs were significantly increased in bone marrow versus blood. ns: no significant, * *p* < 0.05, *** *p* < 0.001.

Driven by the expansion of MDSCs, which is observed in BM of DLBCL patients, together with the fact that MDSCs are BM resident cells, we investigated if BM MDSCs are differentially presented compared to PB in DLBCL individuals. Notably, total MDSCs, M-MDSCs and PMN-MDSCs all exhibited significantly increased frequencies in BM compared to PB (Figure 3A). However, this was not the case when we addressed the same question regarding the MDSC frequencies healthy individuals’ BM and PB compartments. Specifically, we did not identify significant differences in total and M-MDSCs, except for PMN-MDSCs, which were expanded in BM compared to PB (Figure 3B). Overall, these results unravel a robust increase of MDSCs in the BM of individuals with DLBCL.

### Blood and BM MDSCs did not associated with disease features and outcome

As shown in Supplementary Figure 1, several disease features were tested in univariate analysis in association with known adverse prognostic factors. Specifically, Ann Arbor stage, age, IPI, elevated LDH, cell of origin, response to treatment, and survival showed no significant association with MDSCs frequencies, both total, M-MDSCs and PMN-MDCS.

**Figure 4:**
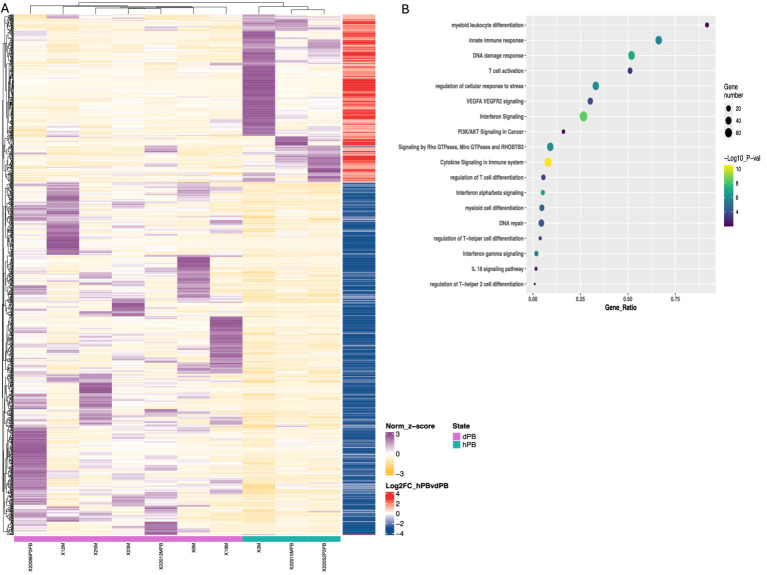
Transcriptomic differences in peripheral M-MDSCs from DLBCL patients and healthy individuals. **(A)** Heatmaps of enriched gene sets found during transcriptomic analysis of cell-sorted isolated M-MDSCs from healthy (green line) versus DLBCL (purple line) individuals. **(B)** pathway analysis of DEFs from M-MDSCs isolated as in **(A)**. Circle size indicate the number of genes/pathway and colour is based on the –Log10 *p* value. Heatmaps are normalized log2 (CPM); counts per million.

### Distinct molecular signatures between BM and peripheral M-MDSCs in DLBCL patients

To shed light on the molecular events and functional properties that underlie the increase of MDSCs in BM and PB of individuals with DLBCL, we sorted highly pure M-MDSCs and subjected them to transcriptomic analysis.

First, to examine how the DLBCL milieu affected the features of M-MDSCs in the periphery, we compared their transcriptome to those isolated from peripheral blood of healthy individuals. As expected, several DEG were revealed, with numerous to be upregulated in DLBCL M-MDSCs compared to healthy M-MDSCs (Figure 4). To this end, genes associate with cytokine and chemokine signaling such as *IL18BP, CCR5, STAT1, IRF8, IL13R1* and *STAT2* were upregulated in DLBCL M-MDSCs while a prominent IFNa/b signaling signature was also observed. Similarly, genes belonging to the myeloid cell differentiation module, such as *CASP8, NOTCH2, IL15* were upregulated in DLBCL MDSCs. Interestingly, gene ontology (GO) analysis pointed to enriched pathways such as “PI3K/AKT signaling,” “regulation of T helper cell differentiation,” “regulation of cellular response to stress” and “VEGFA/VEGFR2 signaling” with numerous genes to be differentially expressed in M-MDSCs from both groups.

**Figure 5:**
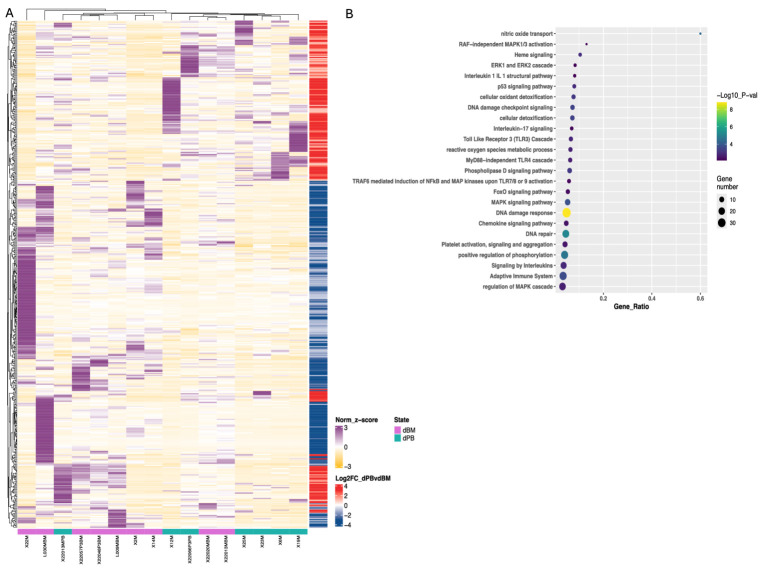
Transcriptomic and pathway analysis of M-MDSCs from blood and BM of DLBCL patients. **(A)** Heatmaps of deregulated genes found during transcriptomic analysis of cell-sorted isolated M-MDSCs from blood (green line) versus BM (purple line) of DLBCL patients. **(B)** pathway analysis of DEFs from M-MDSCs isolated as in **(A)**. Circle size indicate the number of genes/pathway and colour is based on the –Log10 pvalue. Heatmaps are normalized log2 (CPM); counts per million.

**Figure 6:**
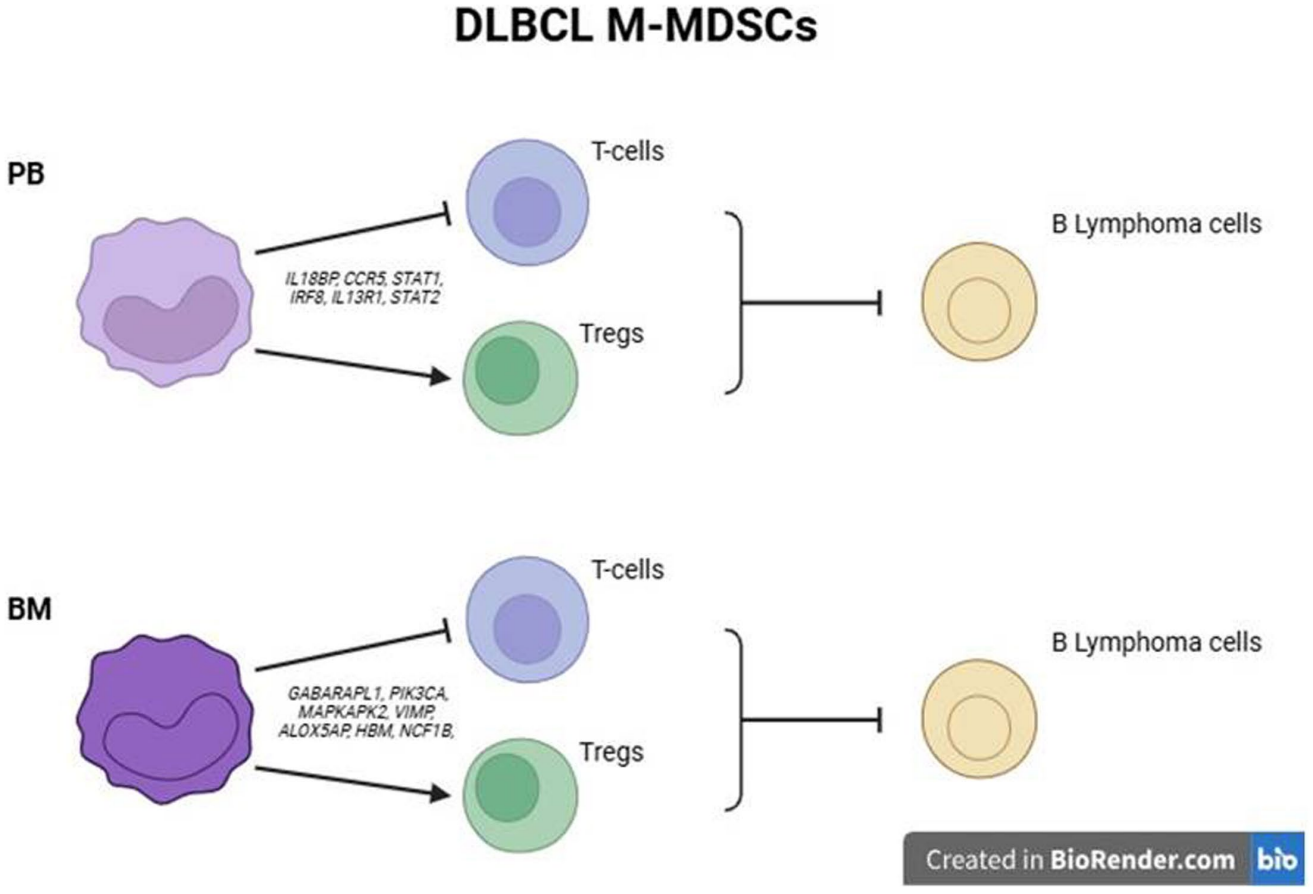
Schematic outline of proposed mechanisms of M-MDSC mediated suppression of DLBCL cells in PB and BM.

Focused on the robust flow cytometry data presented in Figure 3A, we compared the M-MDSC transcriptomes between PB and BM of individuals with DLBCL (Figure 5). Following our analysis, several DEGs were revealed to be significantly upregulated in BM M-MDSCs, which are associated with MDSCs’ functional properties. In detail, genes involved in the regulation of the autophagy pathway such as *GABARAPL1*, *PIK3CA* and *MAPKAPK2,* in cellular oxidant detoxification (i.e., *VIMP*, *ALOX5AP*, *HBM*), heme signaling (*CRTC2, TGS1, HBA2, HBB, HBA1*) and reactive oxygen species metabolic processes (i.e., *NCF1B, DDIT4*) all of which involved in the regulation of MDSC differentiation and function. In contrast, genes that are involved in the IL-17 pathway (such as *RPS6KA5, MAPK1, NFKB1, DUSP6*) and MyD88-independent TLR4 cascade (i.e., *CASP8, 1, DUSP6, UBE2D2, MAPKAPK2*) were significantly upregulated in PB M-MDSCs consistent with increased cytokine signaling in M-MDSCs in the circulating M-MDSCs of DLBCL individuals. In support, gene ontology analysis revealed enrichment in “signaling by interleukins” pathway, as well as “adaptive immune system” pathway. Other pathways such as “DNA damage response,” “p53 signaling” and “platelet activation, signalling and aggregation” were also enriched which may provide new insights in the contribution of M-MDSC in the immunosuppression of DLBCL (Figure 6).

Overall, these findings propose that BM M-MDSCs may exhibit additional mechanisms of immunosuppression to those established by peripheral M-MDSCs, which may contribute to DLBCL disease.

## Discussion

Although an intense immunosuppressive activity of MDSCs has been reported in various hematological malignancies, such as multiple myeloma (MM), acute myeloid leukemia (AML), acute promyelocytic leukemia (APL), B-cell acute lymphoblastic leukemia (B-ALL) myelodysplastic syndromes (MDS), and in chronic myeloid leukemia (CML) (28–30), in lymphomas, the study of MDSCs is rather limited, mostly focused on DLBCL and Hodgkin’s Lymphoma (HL) (34). The majority of studies are focused on circulating MDSCs, while the role of BM-resident MDSCs in these diseases is largely ignored. Herein, through phenotypic and transcriptomic analysis we revealed that BM MDSCs are increased compared to healthy BM and to peripheral DLBCL MDSCs and we identify new mechanisms that underlie the BM resident M-MDSCs in DLBCL patients.

Our findings demonstrate a significant increase of total MDSCs in the blood of DLBCL patients compared to healthy individuals, which reflected a marked enrichment of M-MDSC cell subset while PMN-MDSCs did not reach statistical significance. In line with our findings, a study by Wang et al. described that the levels of M-MDSCs were significantly increased both in newly diagnosed as well as in relapsed DLBCL patients and are associated with tumor progression and overall survival (OS), indicating that the level of M-MDSCs can be used as a biomarker for poor prognosis in DLBCL patients (35). Similarly, Wu et al., evaluated the percentage of whole blood M-MDSCs in 144 newly diagnosed patients and confirmed their prognostic value, while circulating M-MDSCs decreased significantly after R-CHOP treatment (36). Of interest, in a recent study by Azzaoui et al., both the M-MDSCs (CD14^pos^HLA-DR^low^) and PMN-MDSCs (Lin^neg^ HLA-DR^neg^CD33^pos^CD11b^pos^) were significantly increased in blood of 66 newly diagnosed DLBCL patients compared to 45 healthy donor samples (37). M-MDSCs were increased in high risk (HR) patients with an age-adjusted IPI ≥ 3 and were associated with a worse event-free survival (EFS), while PMN-MDSCs did not correlate with any risk factors or EFS. This is contrast to our results in regards to PMN-MDSCs since, in our study, they were not significantly altered in periphery of the DLBCL cohort. A possible explanation for this discrepancy could be the definition of PMN-MDSCs since we included CD15 for their characterization. The method of isolation of MDSCs, could also generate an impact on the results. Indeed, several caveats exist in the study of MDSCs not only in the field of DLBCL but in general, that limits the reproducibility of the results. For example, some studies have analysed MDSCs and subsets in whole blood, while others have constructed experiments in isolated mononuclear cells. This may affect the frequencies of MDSCS, particularly PMN-MDSCs. Furthermore, despite the efforts for the use of a unified system to phenotypically characterize MDSCs and their subsets by flow cytometry (38) still, this is partly followed, thus resulting in non-reproducible results. Therefore, to better characterize the peripheral MDSCs in DLBCL patients and to provide mechanistic insights, we performed transcriptomic analysis and compared to peripheral MDSCs from healthy individuals. As expected, genes involved in inflammatory pathways and particularly in cytokine signalling, with emphasis in type I IFNs, were highly upregulated in DLBCL M-MDSCs. Type I IFNs are closely linked to the immunosuppressive function of myeloid cells in cancer (39). The DNA damage response pathway was enriched in disease M-MDSCs suggesting that possible conditioning by the inflammatory mediators of the disease induces stress in the BM, activating the DNA damage response. Support for this hypothesis was obtained since genes that belong to “regulation of the cellular response to stress” were also enriched in M-MDSCs of DLBCL patients. The DNA damage pathway was previously reported to be upregulated in peripheral MDSCs from patients with colorectal cancer (40). Finally, the expression of genes that belong to myeloid cell and T cell differentiation were enriched in DLBCL M-MDSCs, which is consistent with functional properties of MDCSs in cancer (26, 41).

MDSCs are generated and reside in the BM, while they exit in the periphery in response to pathological insults. Notably, during disease, MDSCs expand in the BM, followed by mobilization and further expansion in blood and peripheral lymphoid organs, mainly spleen. In addition, conditioning by inflammatory mediators as well as therapeutic regimens also could affect BM MDSCs (17). However, the extent to which the BM dictates the functional properties of peripheral MDSCs remains largely unknown. Importantly, most MDSC-related literature has extensively studied peripheral MDSCs, while the BM counterparts have not been investigated, including the field of DLBCL. We attempted to shed light on this by assessing the frequencies and transcriptomes of BM MDSCs in DLBCL individuals compared to peripheral blood and also to MDSCs from healthy individuals. Interestingly, both subsets of MDSCs showed a significant increase in the BM compared to PB of DLBCL patients. To understand the molecular mechanisms that operate in BM MDSCs, we isolated M-MDSCs and subjected them to transcriptomic analysis. Isolation of PMN-MDSC was technically challenging and we did not proceed with this subset. Several genes involved in the nitric oxide pathway and the reactive oxygen species process, both of which constitute major immunosuppressive mechanisms of MDSCs, were upregulated in BM MDSCs (42). This indicates that MDSCs are already primed in the BM of DLBCL patients to release suppressive mediators. In support, autophagy genes were also upregulated in BM MDSCs, and autophagy has been shown to influence the suppressive function of M-MDSCs (43). In contrast, expression of genes involved in signaling by interleukins, such as IL-17 and IL-1, were upregulated in PB compared to BM MDSCs. This agrees with literature highlighting the importance of IL-17 in mediating the immunosuppressive function of MDSCs in the periphery to promote tumor development (32, 44).

Notably, the levels of MDSCs in DLBCL have been also correlated with response to treatment. To this end, in a recent study (45), 31 DLBCL patients were prospectively evaluated for M-MDSC and PMN-MDSC distribution using flow cytometry from peripheral blood collected before and after chemo-immunotherapy and at the time of relapse. Patients with DLBCL clustered into three distinct immunotypes according to MDSC levels and subtype predominance: M-MDSC high, PMN-MDSC high, and MDSC low, and correlated with DLBCL subtypes. The M-MDSC high immunotype was associated with the germinal center B cell−like (GCB) subtype and PMN-MDSC high with the non-GCB subtype. Chemo-immunotherapy partially reduced M-MDSC distribution, while persistence of PMN-MDSCs after treatment was associated with early relapse. In this line, in relapsed/refractory (R/R) DLBCL, circulating MDSCs and Tregs were found to be increased compared with a healthy control group in a group of 79 patients not eligible for ASCT in the R2-GDP-GOTEL study (46). MDSCs levels were re-evaluated at cycle three and at the end of induction therapy. Only patients with a significant decrease of MDSCs after treatment reaching the levels of the healthy group, had the longest OS. In contrast, based on our findings, the frequencies of total MDSCs as well as M- and PMN-MDSCs did not correlate with any known adverse prognostic factor, such as age, IPI, stage, LDH, COO, response to therapy, or survival. Several reasons may explain these results. The relatively small number of analysed cases, the short follow-up time and the strict criteria for MDSCs immunophenotypic characterization may affect our findings. Furthermore, in the aforementioned studies, there are not any consistent associations, since MDSCs have been correlated with various prognostic factors, suggesting that probably the prognostic significance of MDSCs requires further evaluation or that the percentage of MDSCs cannot be used alone as prognostic factors but rather in conjunction with features of other immune cells, such as Tregs, and expression of immune checkpoint molecules in T-cells (47).

To conclude the findings presented here reveal that MDSCs in the BM of DLBCL patients hold a distinct transcriptomic profile compared to peripheral MDSCs, which may translate to differential immunosuppressive mechanisms exhibited by BM resident MDSCs. Delineation of the mechanisms that operate in the different anatomic compartments and orchestrate the tumor immune escape, will facilitate the design of rationale therapies for DLBCL patients.

## Data Availability

The original contributions presented in the study are publicly available. This data can be found here: https://www.ncbi.nlm.nih.gov/geo/query/acc.cgi?acc=GSE287333.
