# Supplementary material for: Myeloid-derived suppressor cells exhibit distinct characteristics in bone marrow and blood of individuals with diffuse large B-cell lymphoma
Source: Front Med (Lausanne). 2025 Jan 29;11:1515097. doi: 10.3389/fmed.2024.1515097 (PMC11814433; doi:10.3389/fmed.2024.1515097)
Supplement: Supplementary file 1 [file Data_Sheet_1.PDF]

Supplementary Material

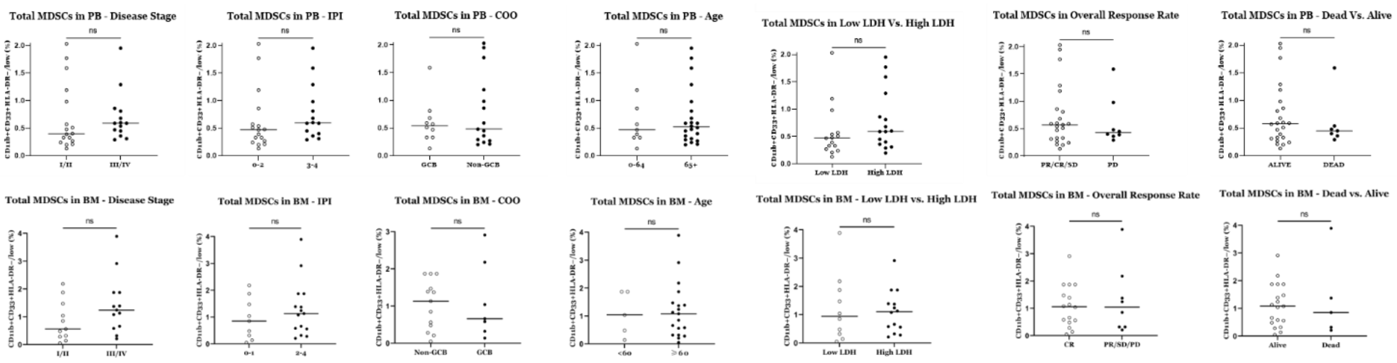

Supplementary Figure 1. Correlations of MDSCs frequencies with disease characteristics and outcome. Frequencies of blood and bone marrow MDSCs did not corelate with disease characteristics, such as stage, IPI, cell of origin (CCO), age, elevated LDH, response to treatment or survival. ns: no significant
